# Right Atrial Thrombus Complicating LVAD Candidacy: A Clinical Case Study

**DOI:** 10.3390/jcm14176242

**Published:** 2025-09-04

**Authors:** Cetin Alak, Aarti Desai, Abdallah El-Sabbagh, Daniel Yip, Rohan Goswami

**Affiliations:** 1 Division of Heart Failure and Transplantation, Mayo Clinic, Jacksonville, FL 32224, USA; cetinalak10@gmail.com (C.A.); desai.aarti@mayo.edu (A.D.); yip.daniel@mayo.edu (D.Y.); 2 Department of Cardiovascular Diseases, Mayo Clinic, Jacksonville, FL 32224, USA; elsabbagh.abdallah@mayo.edu

**Keywords:** right atrial thrombus, thrombectomy, heart failure, LVAD, transplant

## Abstract

**Background**: Right atrial thrombus (RAT), though rare, carries a high risk of pulmonary or systemic embolism and a 25–44% mortality rate. In LVAD candidates, coexisting RAT significantly complicates perioperative management due to their critical condition and high risk of decompensation during surgery. Percutaneous thrombectomy offers a less invasive, lower-risk alternative to open surgery, enabling faster recovery. **Case Presentation**: We present a case of a 76-year-old male with ischemic cardiomyopathy and heart failure secondary to Adriamycin chemotherapy with an ejection fraction (EF) of 26%, diabetes mellitus, and stage 3 chronic kidney disease, scheduled for left ventricular assist device (LVAD) implantation. Preoperative echocardiography revealed a large, multilobulated, mobile thrombus in the right atrium. Given the high surgical risk and thrombus mobility, percutaneous thrombectomy using the AngioVac system (Angiodynamics, Latham, NY, USA) was performed. The thrombus was successfully removed, and the patient was discharged on apixaban for long-term anticoagulation. Advanced medical therapy and transplant/durable LVAD evaluation were delayed by 35 days to allow for the stabilization of any postoperative effects. **Conclusions**: This case underscores the efficacy of percutaneous thrombectomy as a bridge to LVAD in high-risk patients, reducing thromboembolic complications and supporting safe LVAD implantation.

## 1. Background

Right atrial thrombus (RAT) is an uncommon but serious condition that can lead to complications such as pulmonary and systemic embolism. Autopsy studies have found RAT in about 3.1% of cases, often without symptoms [1]. Early mortality rates for RAT range from 25% to 44%, depending on the thrombus’s size and mobility [2].

Management options for RAT include anticoagulation, thrombolytic therapy, surgical embolectomy, and percutaneous thrombectomy. Advanced treatments like thrombolytic therapy and surgical embolectomy have lower mortality rates (14%) compared to anticoagulant therapy (30%). The choice of treatment depends on the patient’s health status and the characteristics of the thrombus [3]. Candidacy for advanced heart failure therapies in patients with right atrial masses may be delayed until appropriate management is undertaken. Differential diagnosis should include metastatic masses, benign or malignant cardiac tumors (such as myxoma, lipoma, or angiosarcoma), thrombus, and vegetation.

Patients scheduled for left ventricular assist device (LVAD) implantation with coexisting RAT pose significant management challenges due to their critical condition and high risk of decompensation during surgery. Minimally invasive procedures such as percutaneous thrombectomy provide an effective alternative to open-heart surgery, with lower mortality rates and early recovery.

This case report illustrates the successful use of percutaneous thrombectomy to manage a large RAT in a high-risk patient awaiting LVAD implantation, highlighting the importance of tailored, multidisciplinary approaches in these complex cases.

## 2. Case Presentation

A 76-year-old male was admitted to our facility with a history of ischemic cardiomyopathy and heart failure secondary to Adriamycin chemotherapy for Hodgkin’s disease (10 cycles of Adriamycin chemotherapy over one year, last treatment was more than 3 years ago), in remission at the time of assessment. Hodgkin’s Lymphoma was diagnosed 18 years prior (initially treated with R-CHOP regimen). Additional history includes coronary artery bypass grafting 8 years prior, diabetes mellitus (HbA1c 7.3%), and chronic kidney disease stage [3]. His baseline left ventricular ejection fraction (EF) was 26%, and he had a single-chamber intracardiac defibrillator (ICD) implanted two years prior for primary prevention of ventricular arrhythmias. He was initially seen in 2021 but did not want to pursue LVAD at that time. One month prior, the patient was admitted for acute decompensation of chronic heart failure with reduced ejection fraction and found to have refractoriness to oral GDMT. He was initiated on Dobutamine 5 mcg/kg/min and remained on that as a bridge to decision. He was readmitted to our facility for a planned LVAD implantation after goals of care discussions were undertaken as an outpatient one month later. His at-home medications include aspirin 81 mg QD, atorvastatin 40 mg QD, empagliflozin 10 mg QD, semaglutide 1 mg SQ weekly injections, bumetanide 1 mg QD, and dobutamine 5 mcg/kg/min. Liver function tests, oxygenation, inflammatory markers, and coagulation profiles were within normal ranges.

**Figure 1:**
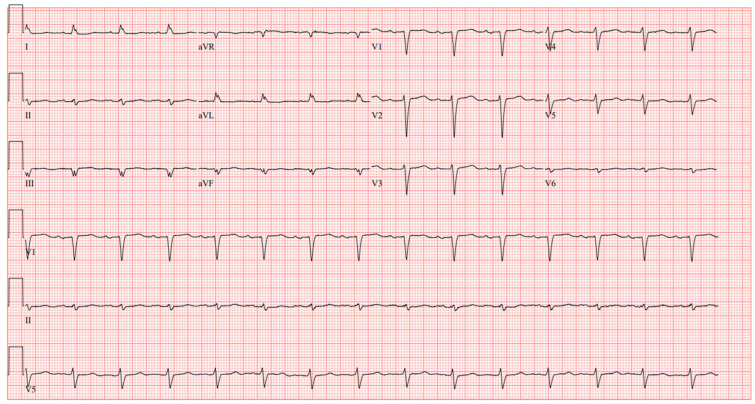
12-Lead surface ECG showing sinus rhythm and poor R wave progression.

**Figure 2:**
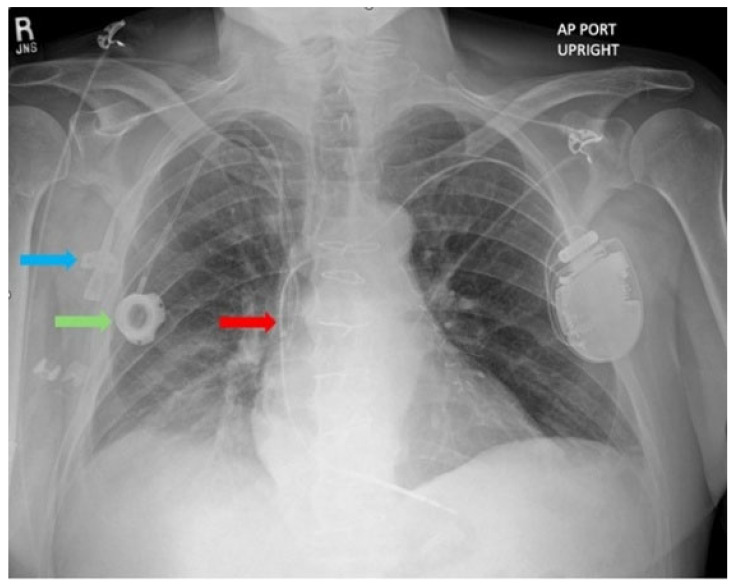
Chest X-Ray. Blue arrow: Peripherally inserted central catheter; Green arrow: Central venous catheter; Red arrow: Single chamber intracardiac defibrillator lead.

On Dobutamine, he showed stable hemodynamics with blood pressure 81/65 (70) mmHg, HR 79 bpm, respiratory rate 18/minute, temperature 36.4 °C, and SaO_2_ 97%. His ECG showed sinus rhythm and poor R wave progression (Figure 1). Laboratory findings included a hemoglobin level of 11.8 g/dL, platelet count 160,000/µL, INR 1.4, creatinine 2.33 mg/dL, glomerular filtration rate (GFR) of 28 mL/min/1.73 m^2^, WBC 8900/µL, procalcitonin 0.12 ng/mL. Chest X-ray revealed a peripherally inserted central catheter (PICC) line, a prior central venous catheter (Port-A-Cath) inserted a month prior, and a single-chamber ICD lead positioned within the superior vena cava (SVC) (Figure 2). Routine echocardiography was performed to evaluate left and right ventricular function, valvular function, and to rule out cardiac masses and thrombus, determining the patient’s eligibility for LVAD implantation. He was deemed ineligible for a heart transplant considering his age, history of prior sternotomy, and Hodgkin’s Lymphoma in remission. Incidentally, echocardiography revealed a large, multilobulated, mobile thrombus measuring 35 × 30 mm in the right atrium (RA) of unclear origin. Further echocardiographic findings showed RA pressure 15 mmHg, RV systolic pressure 29 mmHg, and TAPSE 12 mm, suggesting only moderate RV dysfunction, an LVEF 17%, LVOT TVI 11.6 cm, Cardiac Output 4.1 L/min, Cardiac Index 2.09 L/min/m^2^, a mildly enlarged right ventricular chamber, moderately reduced left ventricular systolic function, and moderate tricuspid valve regurgitation. Elevation in RA pressure was felt to be increased in the setting of moderate TR and overestimates true filling pressures. No thrombus was detected in the other cardiac chambers. Upper and lower extremity Doppler ultrasound performed to investigate the source of the right atrial thrombus showed no evidence of deep vein thrombosis.

The potential etiologies for the RAT in this patient include a medical history of malignancy and heart failure, along with the presence of multiple catheters in the SVC. Pulmonary CT angiography was deferred due to impaired kidney function and concern for contrast-induced nephropathy.

Heparin infusion was promptly initiated, with the dose adjusted based on the anti-factor Xa level. Further treatment options, including anticoagulation, thrombolytic therapy, surgery, and percutaneous thrombectomy, were discussed with the heart team. Consequently, the heart team decided to proceed with percutaneous thrombectomy due to the size and mobility of the thrombus within the right atrium and delay LVAD implantation to mitigate risks associated with possible embolization to the pulmonary arteries, thereby worsening right ventricular strain in the peri-operative LVAD implant period.

**Figure 3:**
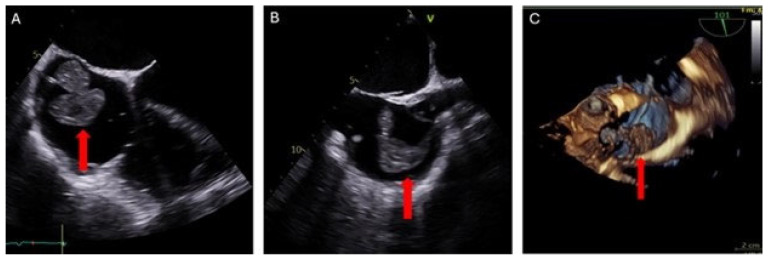
Transesophageal echocardiogram: (**A**) 2D 0° 4-chamber RV-focused view showing right atrial thrombus (red arrow). (**B**) 2D 101° bi-caval view confirming thrombus attachment at the SVC–RA junction (red arrow). (**C**) 3D image view illustrating the size and mobility of the right atrial thrombus (red arrow).

Evaluation was conducted using transesophageal echocardiography (TEE) preoperatively, intraoperatively, and postoperatively. Pre-procedure TEE revealed a large, multilobulated, and highly mobile right atrial mass attached by a thin stalk to the right atrial wall at the SVC-RA junction. The mass measured up to 37 × 31 mm (Figure 3). Moderate mitral valve regurgitation and mild-to-moderate tricuspid valve regurgitation were also noted. A patent foramen ovale (PFO) was not detected.

Informed consent was obtained after thorough discussion of the risks, benefits, and alternatives associated with percutaneous mechanical aspiration thrombectomy of the massive RA thrombus. Subsequently, the patient was transferred to the cardiac catheterization laboratory and placed under general anesthesia with endotracheal intubation.

Access to the venous system was achieved through the right and left femoral veins using 7 French sheaths, respectively. The right femoral vein access was further dilated and upsized to accommodate a 26 French sheath over a stiff wire, while the left femoral vein access was dilated to accommodate an 18 French return cannula. The AngioVac (Angiodynamics, Latham, NY, USA) procedure commenced with the insertion of a 22 French 180-degree AngioVac telescoping cannula system through the 26 French sheath. This cannula was then connected to the veno-venous extracorporeal membrane oxygenation (VV-ECMO) circuit and introduced into the RA. The circuit speeds were ramped up, and the thrombus was successfully aspirated. The patient tolerated the procedure. He was anticoagulated with heparin during the procedure. The patient was extubated and transferred to recovery in a hemodynamically stable condition. The total procedure time was 62 min.

**Figure 4:**
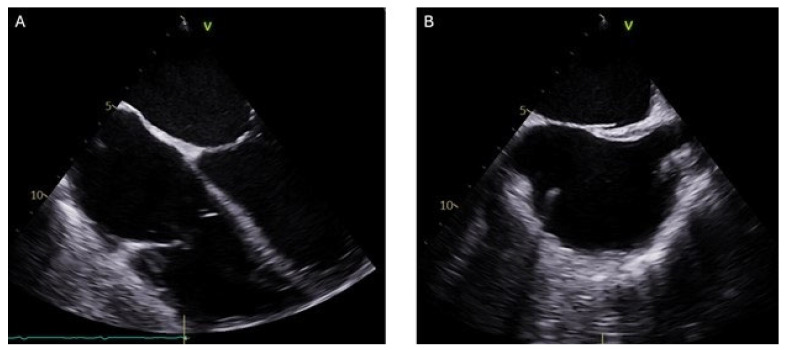
Post-Procedure transesophageal echocardiogram: (**A**) 2D 0° 4-chamber RV-focused view with no residual thrombus. (**B**) 2D 101° bi-caval view confirming absence of thrombus and preserved caval inflow.

Postoperative TEE (Figure 4) and transthoracic echocardiography revealed no evidence of a mass in the RA. However, there was moderate to severe tricuspid valve regurgitation, attributed to a mildly dilated tricuspid annulus and the presence of a device lead. Mild enlargement of the right ventricular chamber size was also noted, along with mild to moderately reduced systolic function. The estimated right ventricular systolic pressure was 36 mmHg, with a corresponding RA pressure of 15 mmHg. No evidence of pericardial effusion was detected.

**Figure 5:**
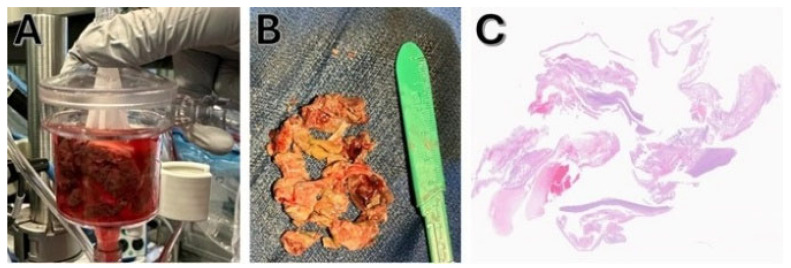
Removed thrombus and histopathological examination: (**A**) Collected clot fragments visualized within the AngioVac filter. (**B**) Aspirated thrombus specimen measuring (5.0 × 4.0 × 1.0 mm). (**C**) Histopathological analysis confirming organized thrombus, the gold-standard diagnosis.

The specimen was retrieved and sent to pathology, where it was identified as multiple irregular portions of gray/tan material measuring 5.0 × 4.0 × 1.0 cm, consistent with thrombus (Figure 5). Following successful AngioVac-assisted thrombus removal, the patient was discharged home with apixaban 5 mg twice daily, dobutamine dose 7.5 mcg/kg/min, and bumetanide 2 mg BD. Other at-home medications included aspirin 5 mg BID, atorvastatin 40 mg OD, empagliflozin 10 mg OD, gabapentin 1 mg weekly, and Ozempic 1 mg weekly. Post-procedure echocardiogram on day 5 showed the ejection fraction improved to 23%, RA pressure reduced to 10 mmHg, RV systolic pressure was 46 mmHg, and TAPSE was 12 cm.

The patient had a progressive decline despite home continuous Dobutamine infusion (7.5 mcg/kg/min) and was deemed out of the window for heart transplant consideration. With progressive renal failure, durable LVAD was considered as an option to improve and optimize end-organ perfusion with the hope of renal recovery.

The patient underwent a successful LVAD implantation nearly 40 days after discharge, but was complicated by biventricular failure and expired shortly after.

## 3. Discussion

The incidence of right atrial thrombus (RAT) remains uncertain, although autopsy studies have identified it in 3.1% of cases, often presenting asymptomatically until complications arise [1]. Despite its rarity, early mortality rates within the first 8 days range from 25% to 44%, contingent upon the thrombus morphology [2]. Our case highlights the unique challenges in patients with end-stage heart failure awaiting advanced therapies such as LVAD placement. Detection of a right atrial mass in patients awaiting LVAD therapy can significantly alter and delay the treatment strategy. In the differential diagnosis of right atrial masses, thrombus, vegetation, and tumors should be considered. Multimodality imaging techniques play a crucial role in differential diagnosis, but the gold standard for diagnosis is histopathological examination [4].

### 3.1. Comorbidities, Risk Factors, and Early Diagnosis

In our patient, a history of malignancy and the presence of multiple catheters in the SVC raised the possibility of thrombus, vegetation, or metastatic masses as potential etiologies for the RAT. The absence of clinical signs of infective endocarditis, the remission status of the patient’s malignancy, and the echocardiographic finding of the mass most likely originating from the catheter-lead in the SVC supported acute formation of thrombus as the leading diagnosis. In such patients, AngioVac-assisted thrombectomy served as a bridge to LVAD therapy and provided the opportunity for histopathological examination, which is the gold standard for diagnosis, thereby playing a critical role in early diagnosis and management.

The differential diagnosis of right atrial masses includes thrombus, tumor, and vegetations, and multimodality imaging can be helpful for further characterization. In our patient, several predisposing factors, including advanced heart failure, the presence of a port catheter, a peripherally inserted central catheter, and an ICD lead, may have contributed to the formation of a thrombus. Echocardiographic follow-up over time consistently supported this impression. Cardiac MRI or PET scans may have added diagnostic value, but these modalities were clinically limited due to the patient’s elevated baseline creatinine and comorbidities, which increased the risk of contrast-based complications. Importantly, AngioVac aspiration with subsequent histopathological confirmation established the definitive diagnosis, making additional imaging unnecessary in this case.

### 3.2. Management Strategies: Anticoagulation, Continuous Monitoring, and Outcome

Management options encompass anticoagulant therapy, thrombolytic therapy, surgical intervention, and percutaneous embolectomy. In the literature, advanced treatments, including thrombolytic therapy, catheter-based interventions, and surgical embolectomy, exhibit a combined mortality rate of 14%, contrasting with anticoagulant therapy’s 30%, showcasing their superior efficacy [3]. Studies suggesting surgical intervention’s effectiveness compared to other advanced therapies note a selection bias towards younger, lower-risk patients chosen for surgery. Conversely, certain studies report percutaneous embolectomy procedures’ success rates for high surgical risk patients with right-sided masses reaching as high as 94.1% [5].

Definitive data regarding the treatment and management of patients with right-sided thrombus planned for LVAD implantation are lacking in the literature. Managing patients with RAT, particularly those deemed at high surgical risk requiring a second surgery for LVAD therapy, poses significant challenges. In such scenarios, alternative approaches such as percutaneous embolectomy modalities can serve as a bridge to LVAD.

For patients planned for long-term LVAD therapy and deemed at high surgical risk, RAT management presents considerable challenges. Conducting routine echocardiography before LVAD implantation is imperative to assess thrombus presence and consider alternative strategies to mitigate surgical risks. Percutaneous thrombectomy effectively addresses the thrombotic burden and avoids complications associated with open-heart surgery, while also minimizing risks associated with additional surgical interventions of LVAD implantation or heart transplant. By directly targeting and extracting intracardiac thrombi, percutaneous thrombectomy provides tailored management for high-risk patients, enhancing treatment precision and patient outcomes. Addressing underlying risk factors for thrombus formation is pivotal in optimizing outcomes for patients requiring long-term LVAD therapy.

The challenges faced in this case include the inability to perform pulmonary CT angiography due to concern for worsening stage 3 CKD. While thrombolytic therapy could have been considered as an alternative treatment option, it was not administered due to the highly mobile nature of the thrombus. The decision to prescribe apixaban for long-term anticoagulation therapy instead of warfarin was made, but comparative efficacy and safety data between the two agents were not available for direct comparison in this specific population. Warfarin was not selected for our patient’s underlying renal dysfunction, comorbidity burden, and the practical challenges of INR monitoring. Recent evidence demonstrated that apixaban is non-inferior to warfarin in achieving thrombus resolution in patients with left ventricular thrombus after myocardial infarction, with a lower risk of major bleeding [6]. Furthermore, observational data from patients with left atrial appendage thrombus support DOACs for achieving earlier thrombus resolution compared with warfarin. Given the pathophysiological similarity between left- and right-sided intracardiac thrombi, we considered apixaban a reasonable and evidence-supported alternative in this high-risk patient [7,8]. Further research and clinical studies are needed to explore optimal management strategies for patients with similar clinical presentations and comorbidities.

The outcome, despite a successful LVAD implantation, was not favorable in our case, and the patient died from progressive biventricular failure. This underscores the complexity of patient selection in advanced heart failure, particularly in patients with borderline right ventricular function and significant comorbidities. Predictive risk scores exist; however, they may not apply to all patients, specifically those with unique situations of intracardiac thrombi and possibly compromised tricuspid valve function. While alternative strategies such as Bi-VAD implantation, temporary right-sided mechanical circulatory support, or ECMO could theoretically have been considered, in this case, the anticipated risks outweighed the potential benefits given the patient’s overall poor prognosis. Although high risk, survival on chronic Dobutamine infusion is known to be less than that of durable LVAD support, and as such, proceeding with LVAD in this case felt reasonable by our multi-disciplinary team. A key lesson from this case is that despite comprehensive preoperative right-sided assessment, including both advanced echocardiographic parameters and invasive hemodynamics, the value of multidisciplinary review and decision-making remains paramount. These steps are essential for identifying patients who may require biventricular support rather than isolated LVAD implantation, and for optimizing outcomes in complex advanced heart failure patients.

### 3.3. Preventive Measures

Preventive strategies for catheter-related thrombosis are challenging, particularly in patients with cancer and advanced heart failure. Although our patient had several risk factors, including the presence of a port catheter and an ICD lead, there was no baseline indication for anticoagulation, since he had neither atrial fibrillation nor active malignancy. Current guidelines do not recommend routine pharmacological prophylaxis for central venous catheters because of conflicting efficacy data and the potential for bleeding complications [9,10]. Randomized studies in oncology populations have suggested a potential benefit of LMWH or low-dose warfarin in reducing catheter-related thrombosis, but subsequent meta-analyses have failed to confirm this as standard of care [11,12]. In our case, anticoagulation was initiated only after the thrombus was diagnosed and histologically confirmed. This highlights that individualized risk assessment, optimal catheter management, and selective use of surveillance imaging may represent more practical preventive strategies in such complex patients. Future studies should explore whether surveillance imaging or tailored prophylaxis in patients with both malignancy and advanced heart failure could reduce RAT incidence.

### 3.4. Current Updates in the Diagnosis and Management of Right Atrial Thrombus

The management of RAT remains challenging, as no single strategy has demonstrated clear superiority across all scenarios [13,14,15,16,17]. Recent reviews highlight that treatment should be individualized based on thrombus morphology, patient comorbidities, and clinical context. In patients with multiple indwelling catheters or devices, contemporary catheter-related thrombosis emphasizes the importance of prevention, early detection, and individualized therapy [16,17]. For those at prohibitive surgical risk, vacuum-assisted thrombectomy is a valuable alternative in advanced centers. A 6-year single-center series reported high technical success with AngioVac, while a more recent retrospective analysis demonstrated procedural success of 81.8%, survival to discharge of 90.9%, and 72.7% one-year survival [18,19]. The prospective multicenter APEX-AV trial confirmed the safety and efficacy of this aspiration platform, with significant hemodynamic improvements and a low rate of major adverse events in over 120 patients [20]. Additional registry and review data—including applications in right-sided infective endocarditis—reinforce the versatility of aspiration systems across different intracardiac mass etiologies [21,22]. In our patient, these contemporary data support AngioVac as a less invasive, effective option in the setting of catheter-related intracardiac thrombi and inotrope-dependent heart failure, as a strategy for bridging to definitive therapy.

## 4. Conclusions

Intracardiac thrombus has a high mortality rate when found in patients with a low-output state, despite contemporary treatment options. Percutaneous treatments such as AngioVac (Angiodynamics, Latham, NY, USA) can serve as a bridge to LVAD in high-risk patients, offering the potential to reduce perioperative morbidity and mortality.

## Data Availability

The raw data supporting the conclusions of this article will be made available by the authors on request.

## Abbreviations

CT	computer tomography
EF	ejection fraction
GFR	glomerular filtration rate
ICD	implantable intracardiac defibrillator
LVAD	left ventricular assist device
PICC	peripherally inserted central catheter
PFO	patent foramen ovale
RA	right atrium
RAT	right atrial thrombus
SVC	superior vena cava
TEE	transesophageal echocardiogram
VV-ECMO	veno-venous extracorporeal membrane oxygenation
